# A qualitative exploration of the client experience of inter-professional practice in the delivery of ActivePlus: a combined smoking cessation and physical activity intervention

**DOI:** 10.1186/s12913-018-3004-2

**Published:** 2018-03-21

**Authors:** G. A. O’Sullivan, Clare Hanlon, T. Dentry, T. Morris, L. Banting

**Affiliations:** Institute for Health and Sport, Victoria University, PO Box 14428 MC, Melbourne, VIC 8001 Australia

**Keywords:** Inter-professional practice, Smoking cessation, Physical activity, Client experience, Client-centered practice

## Abstract

**Background:**

Research investigating interprofessional practice (IPP) frameworks has predominately focused on the service delivery of IPP or educating practitioners through interprofessional education. Minimal research has addressed client outcomes or the experience of clients with IPP in real world contexts. In this paper, we explore the experience of seven participants in the ActivePlus program, an IPP-based smoking cessation intervention combined with physical activity promotion.

**Methods:**

Participants informed on their program experiences through post-program in-depth interviews. A thematic analysis drew out themes pertaining to participant experiences of the joint practice element of the IPP model of care.

**Results:**

Analysis identified two major themes: the joint practice experience, and the client-centered approach of the IPP model of care. Participants reflected on the ways that having two health practitioners in joint sessions benefited their intervention experience, as well as providing some critical feedback. Participants also reported observing and valuing aspects of client-centered practice that strengthened the rapport within the practitioner-client team and aided their behaviour change progress. The client-centered practice was instrumental in overcoming initial teething issues with joint session delivery and alleviating pre-program participant concerns about being outnumbered by multiple practitioners.

**Conclusion:**

Despite some early teething issues, participants reported a positive acceptance of the IPP and joint session delivery model, which added value to the overall ActivePlus program. Results from this research can provide practitioners with a client perspective on the key aspects they perceive as important in IPP joint session delivery. Further investigation into the client perception in similar interventions is recommended with larger samples and non-clinical groups.

## Background

In Australia interprofessional education (IPE) and collaborative practice were recognised to assist with chronic lifestyle diseases [1]. A year later the World Health Organisation (WHO) launched their framework for action on IPE and collaborative practice, recognising a global health workforce crisis and recommended chronic lifestyle diseases as one of the areas that would benefit from IPP [2]. The framework was based on research evidence showing that IPP can improve access, appropriate use of resources, and health outcomes. The nature and weight of that evidence however was not spelt out [3] and minimal research has been conducted since this time to support these claims.

There is concern that health professionals struggle to understand collaboration [4]. In particular, if roles and boundaries change, then power, status and authority alter and professional socialisation is challenged [4, 5]. In a systematic review of interprofessional health care teams, Micken ([6], as reported in [2]), assessed the outcomes of effective teamwork on organisational, team and individual benefits (including to client and team members). Findings revealed few consistent outcomes.

Client feedback on the value of IPP for the benefit of their health is vital. A recent editorial on the impact of interprofessional education on practice and patient outcomes recommended the inclusion of patient experiences in study designs to better align education and practice and to impact person-centred outcomes [7–10]. The authors also noted substantiating evidence however, for a positive link between IPP and client outcomes, has been scant in the literature. Despite the impact on client outcomes being vital this is where evidence is weakest [11], and studies that sought feedback on their experience recognise that evidence is limited [12].

In a major study of mechanisms of teamwork (one of the key elements of IPP) in stroke care pathways, clients did not notice the teamwork that was meant to be evident between themselves and their team of practitioners [12, 13]. The researchers hypothesized that these clients were more concerned with the care and treatment required by ‘the team’ than the processes of teamwork per se. The lack of evidence on the direct impact of teamwork on clients during IPP warrants further investigation.

### IPP teamwork, physical activity and smoking cessation

An area of health intervention that could benefit from the joint practice element (patient consulting sessions held jointly with multiple practitioners) of IPP teamwork is smoking cessation. In particular, the emerging use of physical activity (PA) behaviour change in conjunction with smoking cessation efforts where the composite benefit addresses two compatible health issues (smoking cessation and PA) in one intervention.

Several community based programs have shown that PA behaviour change can influence the success of smokers attempt to quit with the added benefit of increased levels of PA (e.g., [14, 15]). PA has shown to be an effective adjunct to smoking cessation interventions and can help diminish cigarette cravings [16] and other withdrawal symptoms, such as insomnia [17], weight gain [18], mood disturbance [19] and stress [20]. Furthermore, increases in PA have been shown to improve behavior change self-efficacy [21], and improved self-efficacy has been connected to increased levels of sustained abstinence in smoking relapse prevention programs [22–24]. This suggests that the behavior change self-efficacy gained from increased PA may also empower smoking behavior change.

In a 2014 systematic review of randomized-control-trials for PA based smoking cessation programs, Usher, Taylor and Falkner [15] provided evidence of the effectiveness of such programs. Of the 20 studies reviewed, four-showed evidence of smoking cessation lasting in the short term (3 months), and two showed longer-term effectiveness (6-12 months). In the same year, Taylor et al. [25] reported a large-scale randomized control study that utilized phone and face-to-face counselling sessions to support behavior change regarding smoking behavior and increasing PA. The intervention results reflected the efficacy of this approach with almost a quarter of participants in the intervention arm achieving a quit attempt, while 10% achieved abstinence at 16 weeks and over a third achieved a 50% reduction in daily cigarettes smoked.

The current paper expands the knowledge of smoking cessation intervention programs that also utilise PA promotion, and introduces the IPP model of joint practice, this program is known as ActivePlus. ActivePlus combines the delivery of the Quit smoking cessation program with the services of exercise physiologists in a community health setting. Previous iterations of the ActivePlus program (unpublished pilot studies), revealed that success was attributed to the allied health specialists. Patient feedback however, revealed independent specialist consultations resulted in the client being the mode of communication between practitioners, and the smoking cessation support from Quitline was impersonal, repetitive and reliant on participant initiation in comparison to the ongoing personalised support for PA offered by the exercise physiologist. This feedback inspired the introduction of the IPP model of joint practice into the current ActivePlus program, where both practitioners are present in all consultations to create strong interactions between specialists and ensure complimentary rather than contradictory advice is provided. In this situation, clients openly interact with the practitioners and a team is formed that provides client-centered care, active listening and a shared client narrative, all of which are key elements of IPP [26].

To address the lack of literature on clients’ experience and the direct impact of teamwork on clients when involved in IPP, the present study focuses on the interaction between clients and the practitioner team in the smoking cessation and PA context. Therefore, the aim of this paper is to explore the IPP aspect of the ActivePlus program gained from the experience of participants.

## Methods

### Design

To address the focus of our research, we adopted a qualitative design. Data collection involved semi-structured interviews for 40-60 min with each ActivePlus participant after they stopped or completed the program to explore their experience of the IPP consultation process. Interviews were conducted by the project coordinator (LB) and took place in either the community health centre that housed the project or in cafes at the request of participants. Interview questions focused on the participants history with smoking, quit attempts and PA, and their experiences throughout the 12-week ActivePlus program.

### Participants

Promotional flyers in health service centres and local newspapers about the ActivePlus program were advertised over a four-week period in the municipality where the program was to be conducted. Practitioners within the health service centres also provided program flyers to suitable clients who may be interested in the program. Potential participants then contacted the study co-ordinator (LB) and were provided a study information sheet. After reading the information sheet, potential participants would then contact the co-ordinator to confirm their involvement in the program. Participants were then sent a consent form to complete and bring to the first clinical consultation. The study co-ordinator attended the initial consultation to answer any questions and collect the signed consent forms.

All people who expressed interest and met the inclusion criteria were invited to partake in the intervention. Initially, 10 people were recruited who met the intake criteria that comprised smoking between 15 and 30 cigarettes per day, with the first cigarette within 30 min of waking. Three people did not attend the first session and were not able to be contacted. Five completed the 12-week program while two others progressed more than half way before dropping out. One of the two participants stopped the program because he had quit smoking and the other decided she wasn’t ready to try and quit. These two were interviewed on their experiences and included in the sample. Thus, seven participants were interviewed. This included five female and two male participants, with an age range of 26 to 66 years (3 over 60) and with a smoking history of 10 to 45 years. Therefore the current paper used a purposive sampling method that recruited all participants that undertook the intervention (including the two that almost completed the program).

While seven interviewees is a relatively small sample, several factors influenced the sample size. Challenges to participant recruitment limited the intervention sample size. Recruitment for the program was quite slow and the study had a limited timeline and resources. Typically, recruitment for face-to-face smoking cessation interventions is difficult, with an average success rate of about 2% [27]. Furthermore, recruitment for community based interventions can often be tricky and affected by numerous nuances within the target community [28]. However, smaller sample sizes can be tolerated when it appears that emergent themes have sufficient data and all aspects of the phenomena have sufficient accounts [29]. Patten ([30], pp. 313) argues that “The validity, meaningfulness, and insights generated from qualitative inquiry have more to do with the information richness of the cases selected and the observational/analytical capabilities of the researcher than with sample size”. The current study involved exploratory interviews up to 60 min in duration. While not as in-depth as life-history type research, participants were aloud whatever time they needed to explore their intervention experiences with a skilled and experienced interviewer.

### Intervention

The intervention was conducted from the end of 2015 to the beginning of 2016 in Victoria, Australia. Smoking cessation was the key focus of the intervention and was addressed using Quit advisor strategies implemented by Quit Victoria. PA promotion was addressed through physical activity consultation (PAC) [31]. PAC applies key elements of the transtheoretical model, self-efficacy theory, and self-determination theory within a motivational interviewing framework, which is a client-centered technique, focused on guiding clients to generate their own preferred physical activities with appropriate goals. This enables the client to feel empowered to self-manage and supported to make behavioral changes [32, 33].

The practitioners involved in the program had a background in IPP care and participated in a one-day workshop on IPP principles, including joint sessions, for the purpose of the ActivePlus program. The workshop consisted of a didactic presentation, interactive group work and an interprofessional discussion. Their professional expertise included two exercise physiologists, who were trained in the PAC philosophy and methods, and two Quit trained advisors who were also health care nurses.

Each participant was allocated one exercise physiologist and one Quit advisor. Together they formed a joint consultation/session to collaboratively design the smoking cessation and PA program and work towards the client reducing their intake of cigarettes and increasing their levels of physical activity. Joint sessions allowed for parallel support from each practitioner and each health issue as needed, and gave opportunity for visible teamwork between both practitioners where they could reinforce and build on the strengths of each.

The team met up to five times in joint sessions during the 12-week program and participated in two joint session telephone meetings. Several clients missed one or two sessions.

### Analysis

Interviews were transcribed verbatim and inductive thematic analysis [34] was then conducted by a researcher independent to the intervention program and trained in qualitative methods (GO). The inductive analysis allowed the researcher to be open to codes and themes that evolved with some independence from the structure of the program. A second researcher, trained in qualitative methods (LB), checked the initial inductive codes and themes identified by the independent researcher. The two researchers discussed and agreed on alterations to the codes and themes. This constituted a process of analyst triangulation [30] that contributed to analytical rigor. Due to the limited purposive sample, analytical saturation was not able to be achieved.

In the thematic analysis, we used semantic coding where the explicit meaning of data is described, interpreted and then theorised about the patterns observed [35]. We sought an essentialist/realist type of knowledge [34] from the analysis, which means motivations, experiences and meanings were theorised in a straightforward way from the language in the data. In other words, we looked at the participant reflections in a straightforward manner avoiding over-interpreting the meaning of their words. Entire interviews were coded and themed, however, in this paper we present only themes related to participant reflections of the IPP aspect of the ActivePlus program.

## Results

Seven participants were interviewed to ascertain their experiences with the IPP aspect of the ActivePlus program. Of these seven, only two participants reported at least 150 min of PA per week (recommended minimum for health benefits) at intake. The sample was relatively clinical as all participants were existing clients of the partner health service recruited by internal referral. Participants reported physical health comorbidities and one a mental health comorbidity.

Analysis of participant interviews revealed two major themes: The Joint Session Experience and the Client-Centered Approach. The Joint Session Experience referred to participant first-person experience of a health consultation with the two health practitioners. Four sub-themes evolved. The Two Practitioners sub-theme recognised participant-identified benefits of the two-practitioner interprofessional model. The Negative Pre-impressions sub-theme explored the negative views and concerns participants had of the joint session model pre-program. The Critique sub-theme comprised critical feedback from participants regarding the joint session model and, finally, the Joint Session Consultation versus the Medical Practitioner sub-theme explained the comparisons participants made between the joint session experiences of the ActivePlus program and seeing a medical practitioner for the same health issues.

**Fig. 1:**
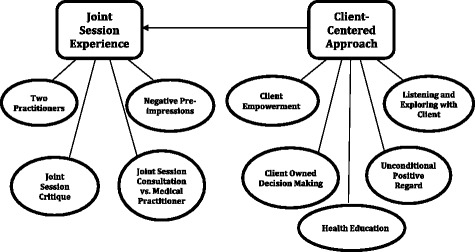
Theme and Sub-theme Map. Rectangles represent themes and ovals represent sub-themes. Interview Guide. Client Participant Interview Guide. Interview guide used to collect data for this study

The Client-Centered Approach theme arose from participant discussions of valued elements of their practitioner consultations that aligned with aspects of client-centered practice. Five sub-themes delineated key aspects of client-centered practice observed and valued by participants. The Client Empowerment sub-theme reflected how participants directed the course of discussions, empowering them to be part of change. Listening and Exploring with the Client was the next sub-theme where participants felt listened to and had time to discuss related issues. The Client Owned Decision Making sub-theme aligned with the principle of the client directing sessions and where participants valued having the decision-making power. The Unconditional Positive Regard sub-theme related to accepting participants for who they were. Health education was the final sub-theme and represented education offered by practitioners that aided participants to make intervention decisions. Figure 1 maps the themes and related sub-themes that arose from the analysis. The arrow represents the contribution the Client-Centered Approach made to the joint session consultations.

### Joint session experience

For most participants, multiple-practitioner consultations represented a new experience. This theme reflected the participants’ experience of the joint session, for example, what it was like, what could be improved, and how it differed from medical practitioner consultations.

One participant’s description of the unique team dynamic experienced in her ActivePlus joint session consultations typified the value participants found in this approach. Instead of potentially feeling outnumbered, this participant experienced a rapport with her practitioners that helped her feel “the centre of attention”. This dynamic also meant she could explore options, address concerns, and ensure she understood health-education due to time given to explore strategies. The participant reported when one health practitioner was making health strategy suggestions, the other would help the participant ask questions on the appropriateness and acceptability of those strategies. She felt supported and this action helped her to feel part of a “team” with her health practitioners.

I hadn’t done anything like that before. It was like being the centre of attention, you felt quite special. I liked it; I thought it was easier to discuss things, because if there was something that [Practitioner 1] suggested that [Practitioner 2] didn’t understand, then she would ask, so that made it easier for me to ask. Because often you go to the doctors and they are good, they always ask if you have any questions, but I never think of all the questions in the session. But having someone else asking made me think like ‘yeah, that’s a good point’, or ‘yeah I didn’t really understand that’. And I also think because we had a whole hour, or even longer sometimes that it was easier because you had time to think about it. (Client 204, 60-69 years).

#### Two practitioners

Participants acknowledged and valued the greater number of tailored strategies on smoking or PA due to the presence of two practitioners in the consultation. They appreciated how these practitioners were open and discussed strategies in front of the participant and felt comforted in the consultation. For example, the practitioner who was not the expert on the topic of conversation would “side with them” in discussions helping them ask questions about suggestions made by the other practitioner. Participants believed it helped move the balance of power away from the professionals and place the client in the centre of their own care. Questions asked by the practitioners demonstrated to participants that they too were learning about the ‘other’ health issue. It became clear to participants that despite one practitioner not being an expert in smoking and the other not an expert in physical activity, yet they were both experts in behavior change. This resulted in both practitioners providing behavior change knowledge and guidance that could be applied to either health issue.

Participants appreciated the clear roles each practitioner had in the consultation. The following response reinforces the feelings from participants.

Yes, I was worried it would be confusing, but it wasn’t, they each had their thing to do and say, so that worked well. And I did feel that when, as an example [Practitioner 3] was talking about smoking because [Practitioner 4] wasn’t an expert, she would think more like me, so [Practitioner 3] would explain to [Practitioner 4] and me why something was a good idea, or why something might help me. Instead of feeling like they would both convince me about things, I felt like I always had someone who was also learning something or think (sic) about something for the first time. [Client 201, 60-69 years].

Participants believed that having two practitioners contributed to the numerous strategies that they, as clients, were provided with, increasing the likelihood that they would find something that worked for them. This was particularly important in cases where the participant was focused on one health issue (e.g., quit smoking) over the other (e.g., physical activity) and the practitioner that did not specialise in that focused issue would contribute by asking questions or providing examples of strategies. Participants believed it helped maintain the rapport of the ‘team’ and prevented one of the practitioners from persisting too much on their health expertise.

… it was good to talk about different things, rather than just smoking or just exercise all the time. And because I went away with lots of ideas to try I felt quite motivated. [Client 204, 60-69 years].

#### Negative pre-impressions

Some ActivePlus participants discussed their initial negative pre-conceived ideas of what IPP was and what joint session consultations would be like. Prior to engaging in the program participants worried that the practitioners would ‘gang up’ against them by having more than one health professional in the room, that it would be confusing, or that the practitioners would be directive about what they had to do. However, without exception these concerns were debunked in practice.

… I was worried they would outnumber me, but that didn’t happen. It did take maybe a couple of sessions to figure out our [errs] rhythm or how it would work, but I think that is normal, even when you just see your GP, sometimes you interrupt each other by mistake. [Client 201, 60-69 years].

I didn’t like it at first. I thought both of them were there to tell me what to do. And I didn’t really know why they wanted me to exercise. I knew that it was part of the program, but I didn’t understand why. So they explained that, and it didn’t seem like there was a rush to finish the sessions. [Client 202, 40-49 years].

#### Joint session critique

Participants identified areas that could be improved within the IPP team. Some participants didn’t see the need for the exercise physiologist’s presence, or that one practitioner would have sufficed. At the same time, however, in these cases, participants noted the ‘non-essential’ practitioner provided other benefits, such as aiding the participant to ask questions or voice concerns.

An initial problem was recognising what team member spoke when. The joint session model of delivery was new to the practitioners and participants, and a period of ‘ironing out the kinks’ and finding the rhythm of who talks when or who answers what questions, was experienced. Participants valued the transparency from practitioners that they too were learning this model of practice and the initial teething issues were not perceived as something that detracted from the program. As explained by two participants:

I was worried that I wasn’t paying enough attention to the [Practitioner 2]. But she was happy enough. She just listened when we spoke about exercise. Actually it was her that probably said that rugby wasn’t a good idea, or sorry, she said that rugby might be tough. I think she has a son too, so she knows that it’s a big commitment, weekend sport. But I thought it was good. [Client 103, 50-59 years].

I think I was the first person they had worked with together. So we all kept talking over each other. Or I would say something and then they wouldn’t know who should respond. But after a while it wasn’t that bad. I was talking more about smoking for most of it, so mostly [Practitioner 1] spoke when we discussed that. But [Practitioner 2] helped me to ask questions. She would sometimes ask things that might be problems for me. She was kind of on my side and she kept always asking if I was happy with things. I liked that. [Client 202, 40-49 years].

#### Joint session consultation versus a medical practitioner

Participants reflected on the contrast between their joint session experience and speaking to a medical practitioner about smoking or physical activity. Participants discussed their experience with the medical practitioner consultation on quit smoking or physical activity recommendations, and on critical judgment made for not being able to quit smoking or increase their physically activity. The joint session consultants, however, were viewed as being more focused on and empowering the client, on providing more time and flexibility for exploring strategies and supportive of behavior change.

For example, one client reflected that when consulting with her doctor she felt “a bit dumb” and not engaged when attempting to identify strategies for smoking cessation and physical activity. She added that the doctor provided directive advice on what she “has” to do, without exploring an appropriate intervention, such as “why she smokes”. This is something she valued in the consultation style of the joint session practitioners.

Another participant explained her preference of the joint session consultation instead of the doctor. These practitioners did not “tell me off” and instead helped foster trust and honesty to assist her identify how to “fix problems” with her behavior change efforts:

…sometimes when you go to the doctor, or the specialist, you always feel a bit like you don’t know what’s happening – a bit dumb. Because they tell you that you have to do this, this, and this. And most of those things you know. Like my doctor, always says, ‘you have to stop smoking’. I know it’s not good for me, but he doesn’t ask why I smoke, or if I want to stop. But [the practitioners] always asked what my reasons were for smoking. And even things like asking if I wanted to smoke less or quit. I thought you could only quit, but they explained that smoking less is also good until you can actually stop smoking altogether. [Client 201, 60-69 years].

#### Client-centered approach

Participants continually emphasised feeling empowered and how the ‘team’ collaborated to identify strategies for their benefit. Many valued aspects of their consultations that were akin to client-centered practice [7–9]. These included feeling listened to, having space to discuss and explore their needs, and owning the decision-making power regarding what interventions they did and did not try.

Participants appreciated the practitioners’ active listening skills. For example, one client noted that her request to not use tablets or patches for smoking cessation was recognised by the practitioners as the topic was not broached again in consultations. This also reinforced the client-centered principle of placing the client in the driving seat of their own treatment:

I think the ladies were very open to my ideas. I was worried that I might be forced to do things that didn’t work for me. Like, I didn’t want to use tablets or the patches. I don’t like the idea of those chemicals and drugs in the body. But I told them that in the first session and then they didn’t mention it again. They really let me say what I wanted to do. [Client 201, 60-69 years].

#### Client empowerment

Participants reported how they valued their practitioners facilitating their ownership of their behavior change. This included participants ‘owning’ their intervention and being supported to gain confidence in their ability to change health behaviors.

For example, one participant reported how the practitioners helped her evolve from wanting to “rely” on a passive intervention option, such as nicotine patches, to being empowered to use her own behavioral and cognitive strategies.

..when I was ready …to try smoking less, they had some ideas. They said that I could try patches. I wanted patches, but they said try without first and see what I can do by myself and I can do some things. I still smoke, but I don’t need to rely on patches. [Client 202, 40-49 years].

#### Listening and exploring with client

Participants reflected on the times they felt listened to and/or had time to explore aspects of behavior change during the joint session consultations. These included face-to-face and telephone consultations. Participants valued the support and solution-focussed technique of exploring life as a non-smoker. For example, one client talked of how his practitioners explored how his behavior change could become realistic based on his perspectives and providing plenty of opportunities to speak:

I would go in and they would say ‘what happened since last time?’ They would ask how much I was smoking, if I had had some cigarettes and why or when. They asked me what I wanted to do, if I wanted to do more exercise or more smoking. They would ask if I had some problems. But they would let me speak and we would talk about how I was doing. [Client 101, 20-29 years].

#### Client owned decision making

The freedom for participants to decide, rather than be prescribed interventions, was a valued part of the consultation dynamic. Many participants reported how the practitioners sought their ideas on what smoking and PA strategies should be attempted. They also valued the ability to decide on what strategies to attempt while not being influenced to try anything they didn’t want to. As explained by these two participants:

They asked what I wanted to do and then we decided. They always said that I could decide. Because sometimes you think they will tell you or teach you all the time. But they were nice. They asked me why I wanted to do things. [Client 101, 20-29 years].

Yeah, they had lots of ideas and then I could choose. One time, just for an example, they said that I could try swapping cigarettes for water. And they say things like that, that you need something in your hand. But for me, I don’t need something in my hand. I need a cigarette. So I said that, and they agreed and then we talked about something else I can try. Because not every idea or plan will work for everyone in the same way, so they were happy with that and I never had to do anything I didn’t want to. [Client 201, 60-69 years].

#### Unconditional positive regard

Participants described their joint session consultations with practitioners as non-judgemental. They felt they were not judged for being indecisive, having relapses, or for not attending a session.

One participant typified this by describing her experience of the ActivePlus program as an overall positive one, largely influenced by the non-judgmental approach of her practitioner team. Her account demonstrates how the elements of the client-centred approach are interwoven – in this case active listening and empowered decision-making power:

It was very nice I must say. I have never been to something like that before. The girls were lovely, very kind natured. I thought they would get frustrated with me, because I know I can be indecisive and sometimes I do worry about things a lot. But they would listen and help me to decide what I wanted to do. I thought it was a very good program. I did like it. [Client 203, 60-69 years].

#### Health-education

Health-education refers to the types of reported education that helped participants understand behavior change regarding smoking and/or physical activity. This included information on smoking delay techniques, how physical activity impacts smoking behavior and physiology, smoking trigger challenge strategies, and the benefits of not thinking in absolutes about quitting (i.e., quitting or nothing).

One example is of a participant who referred to the education she received on the value of reducing smoking before quitting - the need to not think of it as failure - and how this knowledge can help her future quitting behavior. The education provided assisted with her knowing and thinking more about her smoking behavior:

So even if I didn’t quit completely, they said to still think about when I will be ready and now, next time I will have more ideas about smoking, or how it is for me when I quit – what triggers me to start smoking. So I understand more about my smoking now, and I think about it more. I don’t feel guilty when I have one, it’s not that, but I think about it more. [Client 203, 60-69 years].

## Discussion

The focus of this paper is on the clients’ experience of an IPP intervention program. Two major themes emerged from the analysis: The Joint Session Experience and the Client-Centred Approach. Each of these themes was defined by its associated sub-themes (Fig. 1). Findings revealed three components consistently entwined between these sub-themes: the inclusive practices targeted to the client; the change of power differential when the client is working with more than one practitioner; and client-shared decision making.

### Inclusive practices targeted to the client

Findings revealed active listening, a shared client narrative, and a respect for individual beliefs and values, represented the inclusive practices incorporated during meetings by the practitioners and client in the practitioner team. As a result, participants in this research reported feeling “special”, being the centre of positive attention within a group of practitioners, and that it contributed to open discussions about their thoughts and actions on smoking cessation and becoming physically active. These represent elements of client-centered care [7–9, 36].

In addition, the collaborative and inclusive approach between practitioners witnessed by participants during consultations seemed to help build confidence in the joint session approach and contributed to the inclusive environment. This practitioner collaboration seemed to help the practitioners model an inclusive practice that encouraged participants to be drawn into the collaborative dynamic that served as the foundation for the client-centered approach. It also meant that the two practitioners could pool their general behavior change expertise to the benefit of each health issue. These findings provide new knowledge beyond the results reported by Harris et al. [13]. The Harris et al. study did not include deliberate joint sessions and relied on the practitioner teamwork coming across in individual sessions, and as such was not noticed by participants. The ActivePlus findings highlight the benefit of making practitioner teamwork explicitly visible to clients through practitioner joint sessions. Participants not only noticed but highly valued the visible practitioner teamwork.

### Power differential

Findings suggest that educating clients on IPP and working in an IPP team, during the promotional, inquiry and intake stages, may alleviate client fears of being over-powered by multiple health practitioners consulting at one time with the client. Our research identified initial trepidation was evident with some clients, such as fearing being overpowered. After their first consultation with the two practitioners, these fears turned into positive experiences. The implementation of client-centered practices by the practitioners seemed to help re-balance the power away from the practitioners, which benefited client education and behavior change and was preferred to the more directive experiences of general practitioner consultations. Additionally, the presence of two practitioners from two different fields of expertise may have influenced the practitioner-client balance of power. The lack of expertise from one practitioner on issues being raised by the other, sometimes lead the non-expert practitioner seeking the same information as the client.

The valued and successful collaboration by the practitioners in the present study contradicts Reeves and Lewin’s [4] suggestion that health professionals struggle to understand collaboration. Two points may have led to the successful practitioner collaboration reported in the current paper. Firstly, the young age of the practitioners involved in the ActivePlus program, relative to more experienced practitioners, may have affected their perception of their interprofessional power in the consultation [37]. It may also be that the culture of healthcare is changing and recently trained practitioners are more open to joint session collaboration, via IPE, than those trained 20 to 30 years ago [38]. Secondly, all practitioners involved in the ActivePlus program were women, and research suggest that females tend to be more collaborative [39, 40].

The promotion of effective and empowering practitioner collaboration may be a key to the success of the joint session approach. The ActivePlus project educated potential participants about the project’s IPP delivery model throughout the recruitment stages, but several participants still expressed initial concerns about the perceived power held by multiple practitioners in one consultation. Due to the novelty of the joint session approach amongst health service consumers, education and myth busting about the collaborative and client-centered nature of the joint session practice should be part of the service promotion, inquiry and intake phases of client contact. The potential recruitment hurdle of perceived disempowerment in multi-practitioner consultations would be addressed, and clients would also be educated on them being part of a collaborative, client-centred dynamic. The education provided prior to the joint consultation sessions would be timely and may begin to empower clients to understand and make choices about their care [9]. This education may also inoculate clients against the types of experiences reported by participants in the study by Harris et al. [13]. Those participants found the IPP experience confusing and unsatisfactory. It would have been interesting, however, if Harris et al. revisited these clients after a second or third experience to determine whether this confusion was eased with further exposure.

### Client-shared decision making

The need for clients to feel immediately included in the decision-making process was vital from the findings of our research. Participants reported and valued the shared power in consultations and having the final decision about smoking-cessation and PA strategies in their own hands. In this, they felt listened to and empowered to take charge of their own behavior change. Shared decision making is a key principle of IPP and PAC and was stressed in the ActivePlus practitioner training which would have had an influence on the dynamics in sessions. It is also recognised as a powerful and positive aspect of collaboration and may result in decisions by clients that are more acceptable and sustainable than sole practitioner decision making [26, 41].

An important point to note is that some clients may not want, or be capable of, immediately taking on shared decision making about their actions. The clients in our study were capable, however practitioners in a joint session team should aim to recognise clients who may not be comfortable or capable in sharing the decision-making. This caution has also been recognised by Fox and Reeves [42]. Clients, regardless of their ability, can still benefit from active listening and empowerment, while their ongoing capacity to be part of the decision-making process is taken into consideration, within limits (e.g., [43, 44]).

### Interprofessional new knowledge

New knowledge arose from our research that enhances the knowledge of client experience during joint sessions. An example is the value placed by participants on the client advocacy enacted by members of the practitioner team on their behalf. This was perceived as “siding with the client”. It often involved one practitioner asking the other practitioner questions from the client perspective to aid the client’s understanding of education or suggested strategies. This reinforces findings by Harris et al. [13] where clients valued team members who advocated on behalf of the client. The current findings also typify ‘good interprofessional collaboration’ where one profession communicates to another about aspects of clients’ condition that require intervention, that may in less collaborative circumstances not have occurred [45].

Other new knowledge from this study was the importance of effective communication between members of the team. Effective practitioner communication aids several aspects of IPP practice. These include optimising questioning of the other practitioner to gain more knowledge for the client, negotiating when to invite shared decision making, creating effective open dialogue between all members of the practitioner team, and negotiating who answers the general health questions. The current study revealed that clients notice and value when inter-practitioner communication is effective. It also suggested that effective communication contributes significantly to team-client rapport. Group rapport also aided the practitioner team’s recovery from initial adjustments to the joint session approach, which participants saw only as ‘teething’ issues. It is, thus, recommended that the benefits of and strategies for effective inter-practitioner communication should be an essential aspect of practitioner training. The new knowledge gained from the perspective of clients in the current study can assist the advancement of interprofessional education.

### Limitation of the study

Limitations from this study provide opportunities for future research. For example, the participant sample in this program was a relatively clinical one with co-morbidities. This could have contributed to the initial concerns about the joint session approach that were expressed by participants, particularly if participants had experienced judgemental or directive advice from past health consultations. As such, it would be worth investigating the IPP experience in a sample with less complex clinical presentations. Furthermore, the current study involved an intervention that had smoking cessation as the key focus. Utilising the PAC intervention as an anti-smoking mechanism meant that increasing PA became a secondary health goal. Future research should not only investigate the client experience in other health fields, but also with regard to single and multiple health goals.

### Recommendations

Findings from our research showed that clients were appreciative of the two-practitioner model. The practitioners noted how the team members complemented each other and that when there was a ‘non-essential’ team member they still found a way to contribute to the team. The joint session approach would benefit from future research that assesses the client acceptability of various numbers of health practitioners in a joint session consultation. Furthermore, this was a small exploratory study of the first-hand participant experience of seven interviewees in a joint session-based health service. As such, it would be worth conducting larger studies investigating the acceptability of the joint session approach to draw stronger quantitative conclusions regarding its acceptance. The IPP body of knowledge would also benefit from future research structured around the client experience at every stage of IPP delivery, to ascertain what attracts clients to the service and what keeps them going through each stage.

The findings of the current research could also inform IPP practice by describing a practitioner ‘dynamic’ that suggests ways that practitioners from different health fields can come together to form a team. IPP practice would also benefit from our identification of the crucial need for IPP education and myth busting in all the early stages of client engagement. Despite the specificity of the study results to the ActivePlus program, they can none-the-less translate into other health settings. The results demonstrate the benefits of IPP joint practice consultations for health service delivery in community settings, for the addressing of two health issues in combination, for joint sessions particularly involving two allied health professionals, for interventions seeking to use PA promotion as part of an intervention, and for the benefit of smoking cessation type health services.

## Conclusion

From the client perspective IPP and joint sessions added value to the ActivePlus program, aided strongly by effective client-centered practice. The client experience of IPP programs cannot be undervalued, but research in this field is scant. Findings from the current qualitative investigation go some way to filling this gap by suggesting several key elements to an optimal IPP client experience. These elements include the importance of inclusive practices in the IPP consultation, overcoming client concerns of being over-powered by the practitioners, and creating a dynamic of shared decision making. Professionals considering or participating in IPP practice could benefit from exploring the key elements described in this study. Other new knowledge on IPP practice have emerged from this study as have several proposals for future IPP research to address.

## References

[1] Tipp L. Developing Interprofessional Learning and Practice Capabilities within the Australian Health Workforce : A Proposal for Building Capacity within the Higher Education Sector. Learning & Teaching for Interprofessional Practice, Australia (L-TIPP, Aus) Report -Part 1. Broadway: Learning and Teaching for Interprofessonal Practice Project Team; 2009.

[2] World Health Organisation (2010). Framework for action on interprofessional education and collaborative practice.

[3] Barr H (2010). The WHO framework for action. J Interprof Care.

[4] Reeves S, Lewin S (2004). Interprofessional collaboration in the hospital: strategies and meanings. J Health Serv Res Policy.

[5] Mccallin A (2005). Interprofessional practice: learning how to collaborate. Contemp Nurse.

[6] Mickan SM (2005). Evaluating the effectiveness of health care teams. Aust Health Rev.

[7] Rogers CR, Koch S (1959). A theory of therapy, personality and interpersonal relationships as developed in the client-centered framework. Psychology: a study of a science Vol 3: formulations of the person and the social context.

[8] Rogers CR (1957). The necessary and sufficient conditions of therapeutic personality change. J Consult Psychol.

[9] Victorian Depatment of Health (2008). Person Centred practice: guide to implementing person-centred practice in your health service.

[10] Cox M, Cuff P, Brandt B, Reeves S, Zierler B (2016). Measuring the impact of interprofessional education on collaborative practice and patient outcomes. J Interprof care.

[11] Sims S, Hewitt G, Harris R (2015). Evidence of a shared purpose, critical reflection, innovation and leadership in interprofessional healthcare teams: a realist synthesis. J Interprof Care.

[12] Hewitt G, Sims S, Greenwood N, Jones F, Ross F, Harris R (2015). Interprofessional teamwork in stroke care: is it visible or important to patients and carers?. J Interprof Care.

[13] Harris R, Sims S, Hewitt G, Joy M, Brearley S, Cloud G, Drennan V, Greenwood N, Jones F, Kalra L (2013). Interprofessional teamwork across stroke care pathways: Outcomes and patient and carer experience: Final report.

[14] Theodorakis Y, Hassandra M (2005). Smoking and exercise, part ΙΙ: differences between exercisers and non-exercisers. Inq Sport Phys Educ.

[15] Ussher M, Taylor A, Faulkner G. Exercise interventions for smoking cessation. Cochrane Db Syst Rev. 2014;8 10.1002/14651858.CD002295.pub5. 10.1002/14651858.CD002295.pub5 25170798

[16] Roberts V, Maddison R, Simpson C, Bullen C, Prapavessis H (2012). The acute effects of exercise on cigarette cravings, withdrawal symptoms, affect, and smoking behaviour: systematic review update and meta-analysis. Psychopharmacology.

[17] Grove JR, Wilkinson A, Dawson B, Eastwood P, Heard P (2006). Effects of exercise on subjective aspects of sleep during tobacco withdrawal. Aust Psychol.

[18] Farley AC, Hajek P, Lycett D, Aveyard P. Interventions for preventing weight gain after smoking cessation. Cochrane Db Syst Rev. 2012;1:1-149. 10.1002/14651858.CD006219.pub3 22258966

[19] Taylor AH, Biddle S, Fox K, Boutcher S (2000). Physical activity, anxiety, and stress. Physical activity and psychological well-being.

[20] Ussher M, West R, McEwen A, Taylor A, Steptoe A (2003). Efficacy of exercise counselling as an aid for smoking cessation: a randomized controlled trial. Addiction.

[21] McAuley E, Blissmer B (2000). Self-efficacy determinants and consequences of physical activity. Exerc Sport Sci Rev.

[22] Herd N, Borland R (2009). The natural history of quitting smoking: findings from the international tobacco control (ITC) four country survey. Addiction.

[23] Herd N, Borland R, Hyland A (2009). Predictors of smoking relapse by duration of abstinence: findings from the international tobacco control (ITC) four country survey. Addiction.

[24] Prochaska JJ, Hall SM, Humfleet G, Muňoz RF, Reus V, Gorecki J, Hu D (2008). Physical activity as a strategy for maintaining tobacco abstinence: a randomized trial. Prev Med.

[25] Taylor AH, Thompson TP, Greaves CJ, Taylor RS, Green C, Warren FC, Kandiyali R, Aveyard P, Ayres R, Byng R (2014). A pilot randomised trial to assess the methods and procedures for evaluating the clinical effectiveness and cost-effectiveness of exercise assisted reduction then stop (EARS) among disadvantaged smokers. Health Technol Asses.

[26] Légaré F, Stacey D, Brière N, Desroches S, Dumont S, Fraser K, Murray M-A, Sales A, Aubé D (2011). A conceptual framework for interprofessional shared decision making in home care: protocol for a feasibility study. BMC Health Serv Res.

[27] McDonald PW (1999). Population-based recruitment for quit-smoking programs: an analytic review of communication variables. Prev Med.

[28] Wagner EF, Swenson CC, Henggeler SW (2000). Practical and methodological challenges in validating community-based interventions. Children's Serv.

[29] Liamputtong P (2012). Qualitative research methods.

[30] Patton M (2015). Qualitative research and evaluation methods.

[31] Loughlan C, Mutrie N (1996). Conducting an exercise consultation: guidelines for health professionals. J Inst Health Educ.

[32] Miller WR, Rollnick S (2013). Motivational interviewing: helping people change.

[33] Rollnick S, Miller WR, Butler CC (2007). Motivational interviewing in health care: helping patients change behavior.

[34] Braun V, Clarke V (2006). Using thematic analysis in psychology. Qual Res Psychol.

[35] Bryman A (2016). Social research methods.

[36] Morgan S, Yoder LH (2012). A concept analysis of person-centered care. J Holist Nurs.

[37] Burford B, Morrow G, Morrison J, Baldauf B, Spencer J, Johnson N, Rothwell C, Peile E, Davies C, Allen M (2013). Newly qualified doctors’ perceptions of informal learning from nurses: implications for interprofessional education and practice. J Interprof care.

[38] Reeves S, Tassone M, Parker K, Wagner SJ, Simmons B (2012). Interprofessional education: an overview of key developments in the past three decades. Work.

[39] McNair R, Stone N, Sims J, Curtis C (2005). Australian evidence for interprofessional education contributing to effective teamwork preparation and interest in rural practice. J Interprof Care.

[40] Wainer J, Bryant L, Strasser R (2001). Sustainable rural practice for female general practitioners. Aust J Rural Health.

[41] Légaré F, Stacey D, Pouliot S, Gauvin F-P, Desroches S, Kryworuchko J, Dunn S, Elwyn G, Frosch D, Gagnon M-P (2011). Interprofessionalism and shared decision-making in primary care: a stepwise approach towards a new model. J Interprof care.

[42] Fox A, Reeves S (2015). Interprofessional collaborative patient-centred care: a critical exploration of two related discourses. J Interprof care.

[43] Herbert CP (2005). Changing the culture: Interprofessional education for collaborative patient-centred practice in Canada. J Interprof Care.

[44] Légaré F, Witteman HO (2013). Shared decision making: examining key elements and barriers to adoption into routine clinical practice. Health Aff.

[45] Zwarenstein M, Reeves S (2006). Knowledge translation and interprofessional collaboration: where the rubber of evidence-based care hits the road of teamwork. J Contin Educ Health Prof.

